# Interleukin-17 Expression Positively Correlates with Disease Severity of Lupus Nephritis by Increasing Anti-Double-Stranded DNA Antibody Production in a Lupus Model Induced by Activated Lymphocyte Derived DNA

**DOI:** 10.1371/journal.pone.0058161

**Published:** 2013-03-05

**Authors:** Zhenke Wen, Lin Xu, Wei Xu, Zhinan Yin, Xiaoming Gao, Sidong Xiong

**Affiliations:** 1 Institute for Immunobiology, Shanghai Medical College of Fudan University, Shanghai, China; 2 Jiangsu Key Laboratory of Infection and Immunity, Institutes of Biology and Medical Sciences, Soochow University, Suzhou, Jiangsu Province, China; Northwestern University Feinberg School of Medicine, United States of America

## Abstract

Lupus nephritis is one of the most serious manifestations and one of the strongest predictors of a poor outcome in systemic lupus erythematosus (SLE). Recent evidence implicated a potential role of interlukin-17 (IL-17) in the pathogenesis of lupus nephritis. However, the correlation between IL-17 expression level and the severity of lupus nephritis still remains incompletely understood. In this study, we found that serum IL-17 expression level was associated with the severity of lupus nephritis, which was evaluated by histopathology of kidney sections and urine protein. Of note, we showed that enforced expression of IL-17 using adenovirus construct that expresses IL-17 could enhance the severity of lupus nephritis, while blockade of IL-17 using neutralizing antibody resulted in decreased severity of lupus nephritis. Consistently, we observed an impaired induction of lupus nephritis in IL-17-deficient mice. Further, we revealed that IL-17 expression level was associated with immune complex deposition and complement activation in kidney. Of interest, we found that IL-17 was crucial for increasing anti-double-stranded DNA (dsDNA) antibody production in SLE. Our results suggested that IL-17 expression level positively correlated with the severity of lupus nephritis, at least in part, because of its contribution to anti-dsDNA antibody production. These findings provided a novel mechanism for how IL-17 expression level correlated with disease pathogenesis and suggested that management of IL-17 expression level was a potential and promising approach for treatment of lupus nephritis.

## Introduction

Systemic lupus erythematosus (SLE) is an autoantibody-mediated chronic autoimmune disease characterized by the deposition of immune complexes that contribute to severe organ damage. Lupus nephritis, which occurs most often within five years of lupus onset, is one of the most serious manifestations and one of the strongest predictors of a poor outcome [1]. In lupus nephritis, the pattern of glomerular injury is primarily related to the formation of the immune deposits in situ, which induces the inflammatory response by activation of adhesion molecules on endothelium and results in the recruitment of pro-inflammatory cells [2]–[5]. However, the exact mechanisms that lead to lupus nephritis are still unclear [2], [6]. Thus, identification of crucial effectors which are correlated with disease severity of lupus nephritis would be of great prognostic value, and be helpful for providing targets in treatment of lupus nephritis.

Interleukin-17 (IL-17) is a pleiotropic cytokine that participates in tissue inflammation by inducing expression of proinflammatory cytokines, chemokines and matrix metalloproteases [7]. Recently, accumulating evidence has implicated a potential role of IL-17 in lupus [8]–[10]. An increase of IL-17 production from splenocytes and infiltration of IL-17-associated T cells in kidneys of SNF1 mice were reported [11]. Elevated numbers of IL-17-producing T cells were also infiltrated in the kidneys of patients with lupus nephritis [2], [12]. Of note, laser microdissection-based cytokine analyses showed that elevated expression of IL-17 was correlated with clinical parameters in patients with lupus nephritis [13]. These data implicated a potential role of IL-17 in the pathogenesis of lupus nephritis. However, the correlation between IL-17 expression level and the severity of lupus nephritis still remains incompletely understood.

In our previous study, we demonstrated that compared with unactivated lymphocyte derived DNA (termed as UnALD-DNA), concanavalin A activated lymphocyte derived DNA (termed as ALD-DNA) was capable of inducing an autoimmune disease that closely resembled human SLE manifested by high levels of anti-dsDNA antibodies, glomerulonephritis and proteinuria in SLE-non-susceptible mice, which provided a lupus model to elucidate the SLE pathogenesis [14]–[19]. Here we characterized the association between IL-17 expression level and disease severity of lupus nephritis using the ALD-DNA induced lupus model. Up-regulation of IL-17 was performed using adenovirus construct that expresses IL-17, while in vivo blockade of IL-17 was achieved using neutralizing antibody. We found that management of IL-17 expression effectively modulated the severity of lupus nephritis. Consistently, we revealed that IL-17-deficient (IL-17^−/−^) mice were resistant to development of lupus nephritis. Further, we demonstrated that IL-17 expression level was associated with immune complex deposition and complement activation in kidney. Of interest, we showed that IL-17 was crucial for elevating the generation of anti-dsDNA antibody in lupus. These findings could throw new light on the versatility of IL-17 in SLE pathogenesis, and be helpful for developing therapeutic strategy for treatment of lupus nephritis.

## Materials and Methods

### Ethics Statements

This study was carried out in strict accordance with the recommendations in the Guide for the Care and Use of Laboratory Animals of Shanghai Medical College of Fudan University, and was approved by the Committee on the Ethics of Animal Experiments of Fudan University (Permit Number: FDU20110306). All surgery was performed under sodium pentobarbital anesthesia, and all efforts were made to minimize suffering.

### Mice

Female BALB/c mice between 6 and 8 weeks of age were purchased from the Center of Experimental Animals of Fudan University. The B6 IL-17^−/−^ mice were kindly gifted by Prof. Zhinan Yin and all mice were housed in a pathogen-free mouse colony at our institution.

### ALD-DNA Extraction and Purification

ALD-DNA extraction and purification was performed according to our previously described method [16]–[19].

### Generation of ALD-DNA Induced Lupus Model

Generation of ALD-DNA induced lupus model was achieved according to our previously described method [16]–[19]. Briefly, groups of mice (n = 8) were subcutaneously injected under the dorsal skin with 0.2 ml of an emulsion containing ALD-DNA (50 µg/mouse) in PBS plus equal volume of complete Freund’s adjuvant (CFA, Sigma-Aldrich) at week 0, followed by two booster immunizations of ALD-DNA (50 µg/mouse) emulsified with incomplete Freund’s adjuvant (IFA, Sigma-Aldrich) at weeks 2 and 4. Mice receiving an equal volume of PBS plus CFA or IFA, or UnALD-DNA (50 µg/mouse) plus CFA or IFA were used as controls.

### Anti-dsDNA Antibody Detection

Serological anti-dsDNA antibody was detected using mouse anti-dsDNA antibody ELISA Kit (Alpha Diagnostic International) as previously described [18], [20].

### Urine Protein Measurement

Urine protein was measured with the BCA Protein Assay Kit (Thermo Fisher Scientific) according to the manufacturer’s instructions as previously described [18].

### Study of kidney Sections

The immunofluorescence study of kidney sections was performed using polyclonal anti-mouse IgG-FITC (Sigma) or polyclonal anti-mouse complement C3-FITC (MP Biomedical) as previously described [17], [21]. The intensity of fluorescence was evaluated with the ImageJ software (National Institute of Health) as previously described [17], [22]. The histological signs of lupus nephritis were studied by two pathologists unaware of the immunized animals as described elsewhere [23]. In brief, the extent of the pathological lesions was graded on a semiquantitative scale ranging from 0 to 4 as follows: 0 = normal; 1, a small increase of cells in the glomerular mesanguim; 2, a larger number of cells in the mesangium; 3, glomerular lobular formation and thickened basement membrane; 4 glomerular crescent formation, sclerosis, tubular atrophy and casts. The score for each animal was calculated by dividing the total score for the number of glomeruli observed.

### Treatment with Exogenous IL-17

Adenovirus construct that expresses murine IL-17 (Ad-mIL-17, 10^9^ plaque-forming units per mouse) or the control vector that expresses β-galactosidase (Ad-Ctrl) was used as previously described [24], [25].

### Neutralization of IL-17 in vivo

In vivo blockade of IL-17 was achieved using the neutralizing antibody to IL-17 as previously described [26]. Briefly, groups of mice were injected intraperitoneal with 100 µg/mouse of either anti-IL-17 antibody (R&D Systems) or an isotype control antibody (R&D Systems) 24 h before ALD-DNA immunization and then were given at 3 day intervals for 10 weeks.

### Statistical Analysis

Quantitative data were expressed as the mean ± SD. Unpaired t test and Pearson correlation were used for statistical analyses. A value of P<0.05 was considered statistically significant. All statistical analyses were performed by using SPSS statistical software version 16 (SPSS Inc.).

## Results

### Serological Expression Level of IL-17 was Associated with the Severity of Lupus Nephritis

**Figure 1:**
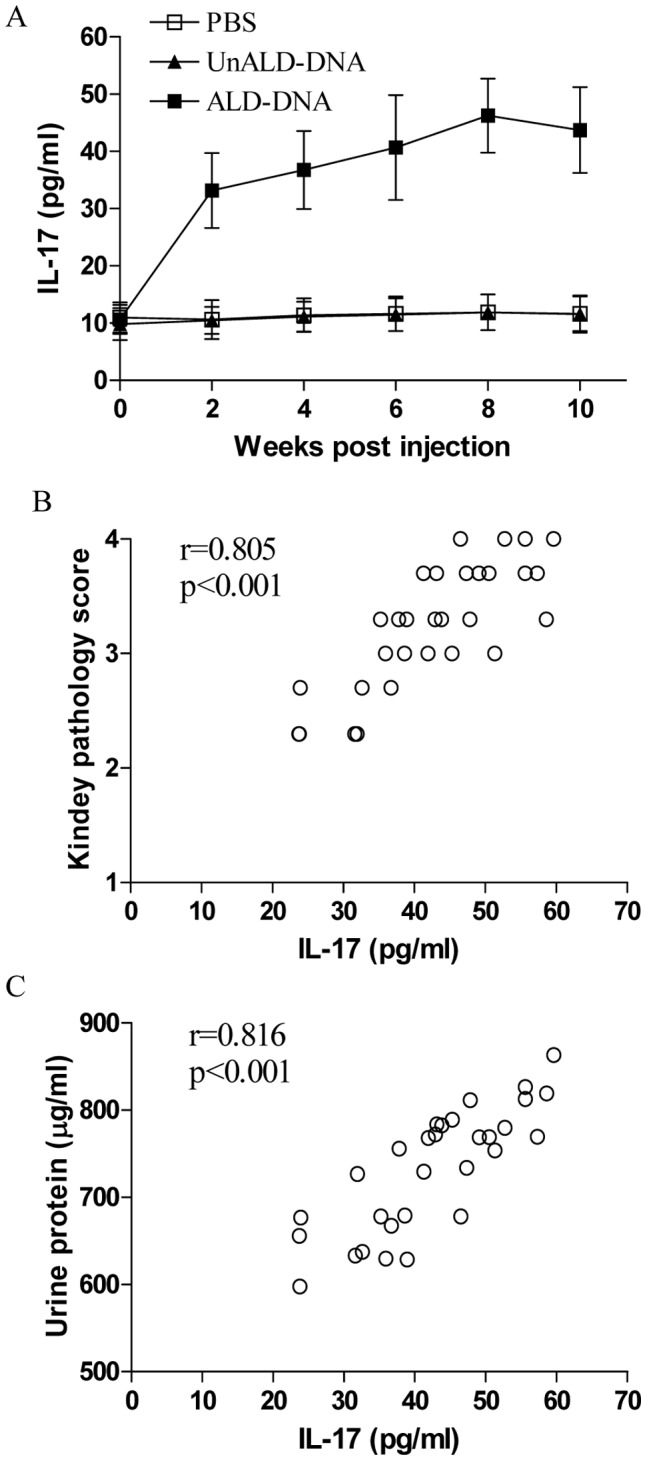
IL-17 expression level was associated with the severity of lupus nephritis. Groups of BALB/c mice were immunized with 50 µg of ALD-DNA as described in methods. (A) The serological level of IL-17 was determined at the indicated time. Data represented the means (±SD) for eight mice in each group. Shown was the represented data from one of four independent experiments. (B and C) The correlation between serological IL-17 expression level and the kidney pathology score, as well as the level of urine protein, was analyzed in thirty two mice eight weeks post ALD-DNA injection. Each dot represented the average value for triplicate in each mouse.

To explore the association of IL-17 expression level and severity of lupus nephritis, we detected the serological level of IL-17 in ALD-DNA induced lupus mice. We found that the serological level of IL-17 was significantly elevated (Figure 1A, p<0.05). Then we analyzed the correlation between serological level of IL-17 and the pathology score of kidney in mice immunized with ALD-DNA for eight weeks. As shown in Figure 1B, we revealed that the IL-17 expression level was closely correlated with the pathology score of kidney (p<0.05). Consistently, we observed that serum IL-17 level was also associated with the level of urine protein in ALD-DNA induced lupus mice (Figure 1C, p<0.05). These data suggested the IL-17 expression level was correlated with disease severity of lupus nephritis.

### Up-regulation of IL-17 Expression Enhanced the Severity of Lupus Nephritis

**Figure 2:**
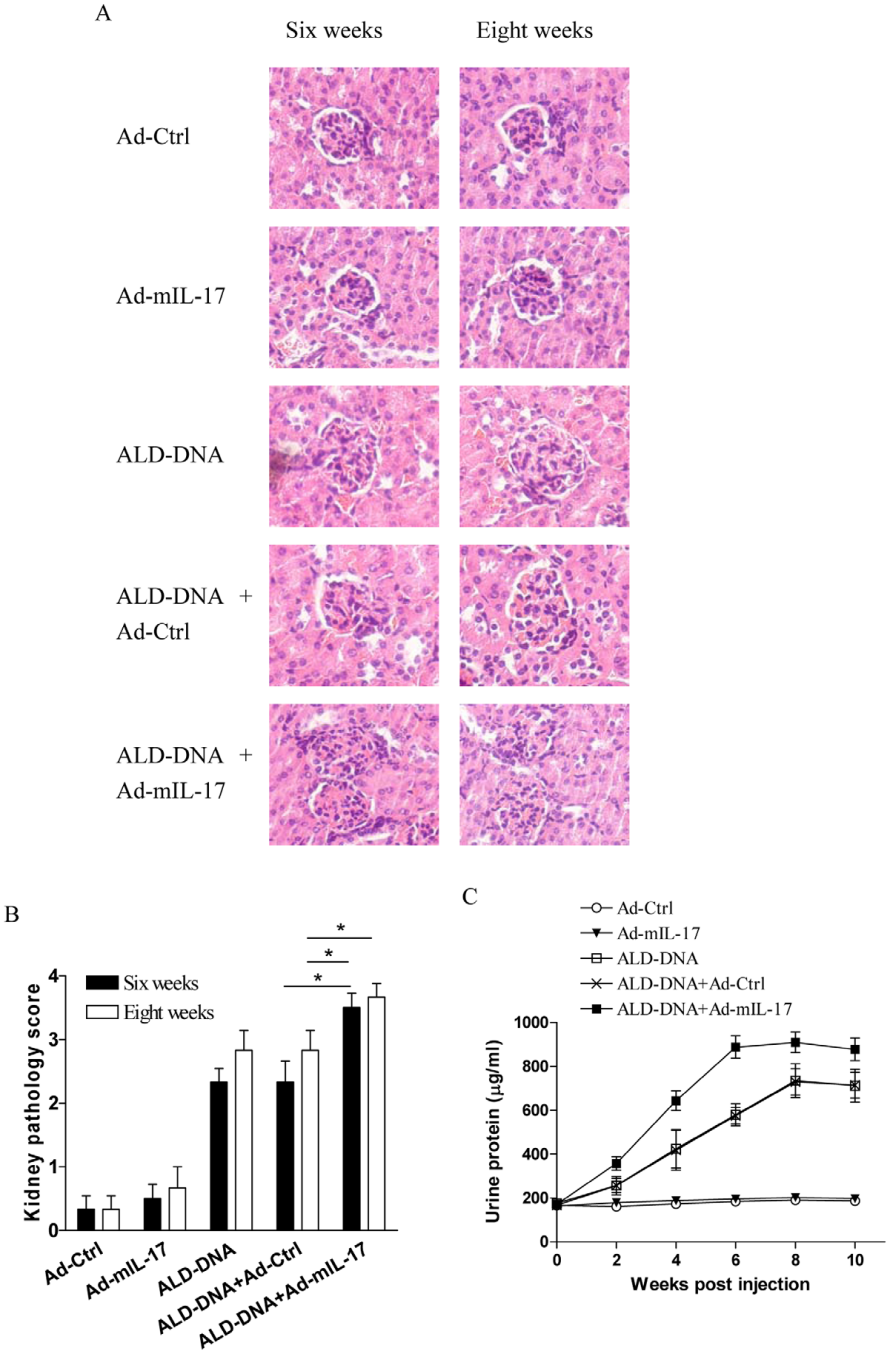
Treatment with Ad-mIL-17 enhanced the severity of lupus nephritis. Groups of BALB/c mice were treated with Ad-mIL-17 or Ad-Ctrl respectively, and then immunized with or without 50 µg of ALD-DNA. The kidney pathology (A and B) and the level of urine protein (C) were analyzed at the indicate time. Data represented the means (±SD) for eight mice in each group. *P<0.05.

To further evaluate the correlation between IL-17 expression level and severity of lupus nephritis, we detected whether up-regulation of IL-17 expression could modify the severity of lupus nephritis. Thus, groups of mice were injected with the adenovirus construct that expresses murine IL-17 (Ad-mIL-17) or with the control adenovirus vector (Ad-Ctrl) respectively, followed by immunization with ALD-DNA. We revealed that the kidney pathology score was significantly higher in mice treated with Ad-mIL-17 than that in control mice (Figure 2A and B, p<0.05). The kidney pathology score in mice treated with Ad-mIL-17 after ALD-DNA injection for six weeks was even higher than that in control mice after ALD-DNA injection for eight weeks (Figure 2A and B, p<0.05). Consistently, we found that the urine protein level in mice treated with Ad-mIL-17 was also significantly elevated compared with that in control mice (Figure 2C, p<0.05). These data demonstrated that up-regulation of IL-17 expression could enhance the severity of lupus nephritis. In addition, we observed that treatment with Ad-mIL-17 alone without ALD-DNA injection resulted in no significant induction of lupus nephritis (Figure 2A, B, C, p>0.05), suggesting that IL-17 was not the startup element for induction of lupus nephritis.

### Blockade of IL-17 Ameliorated the Severity of Lupus Nephritis

**Figure 3:**
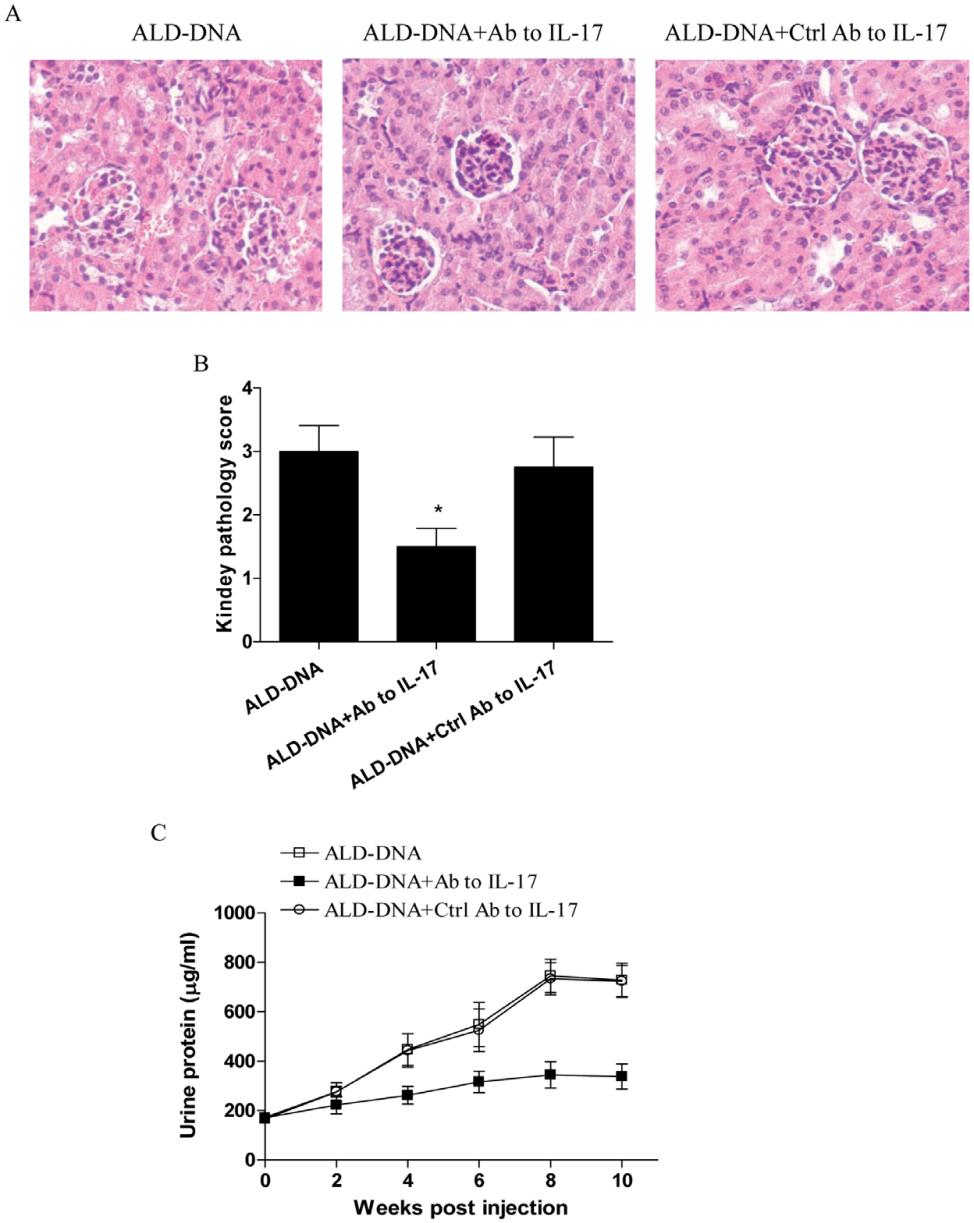
Blockade of IL-17 alleviated the severity of lupus nephritis. Groups of BALB/c mice were injected with neutralizing antibody to IL-17 or the control antibody, and then immunized with 50 µg of ALD-DNA. (A and B) The kidney histopathology and pathology score were analyzed eight weeks post ALD-DNA injection. (C) The level of urine protein was analyzed at the indicated time. Data represented the means (±SD) for eight mice in each group. *P<0.05.

To further detect whether blockade of IL-17 could ameliorate the severity of lupus nephritis, groups of mice were injected with neutralizing antibody to IL-17 and then immunized with ALD-DNA. Eight weeks later, we found that in vivo neutralization of IL-17 significantly abrogated the induction of nephritis (Figure 3A and B, p<0.05). Besides, we revealed that blockade of IL-17 resulted in decreased level of urine protein in ALD-DNA immunized mice (Figure 3C, p<0.05). These data demonstrated that blockade of IL-17 resulted in amelioration of lupus nephritis.

### IL-17^−/−^ Mice were Resistant to Induction of Lupus Nephritis

**Figure 4:**
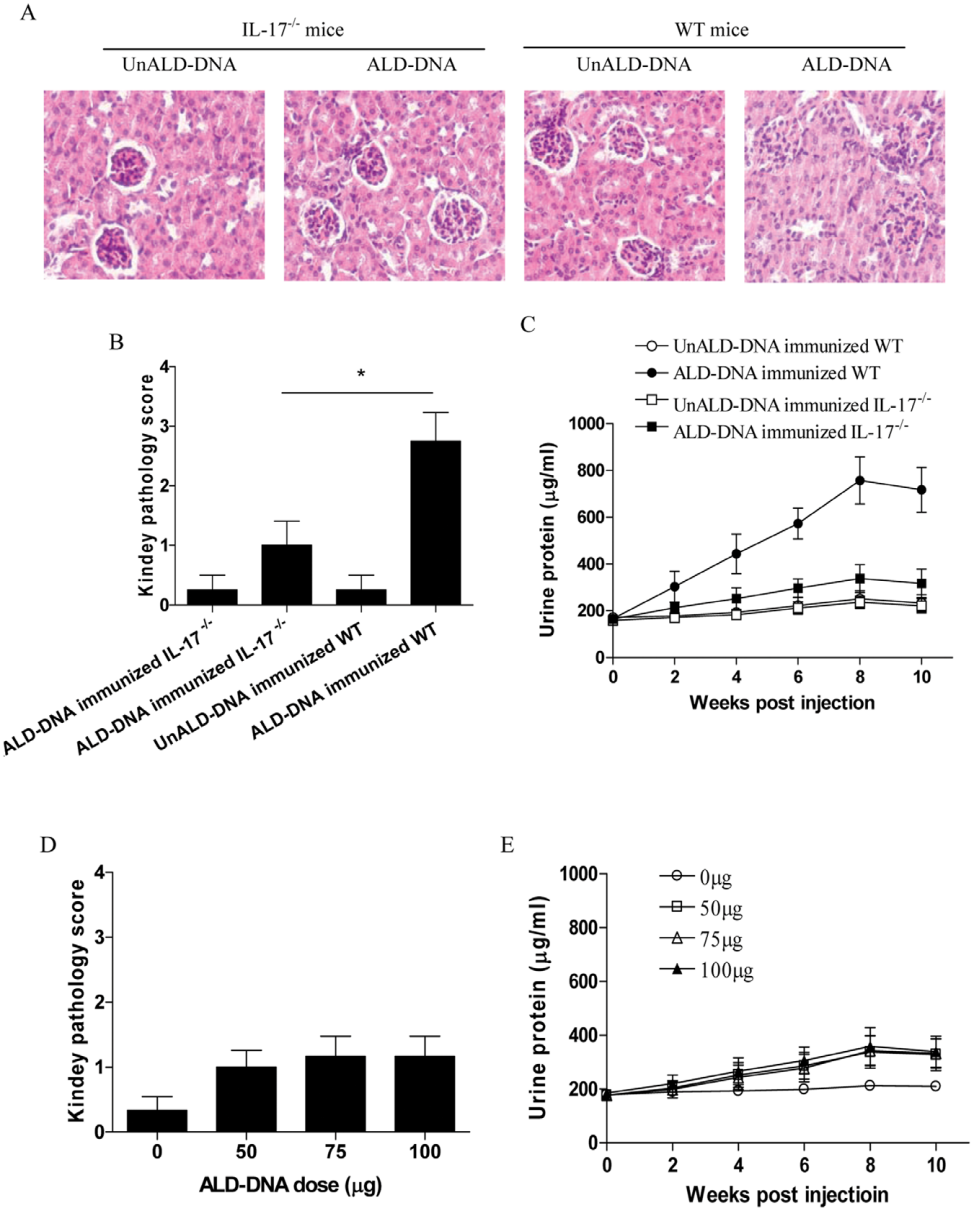
Impaired induction of lupus nephritis in IL-17^−/−^ mice. (A-C) Groups of B6 WT mice or B6 IL-17^−/−^ mice were immunized with 50 µg of the indicated DNA. The kidney histopathology and pathology score were analyzed eight weeks post ALD-DNA injection. The level of urine protein was determined at the indicated time. (D and E) Groups of B6 IL-17^−/−^ mice were immunized with the indicated dose of ALD-DNA. The kidney pathology score was analyzed eight weeks post ALD-DNA injection. The level of urine protein was determined at the indicated time. Data represented the means (±SD) for eight mice in each group. *P<0.05.

To further verify the correlation between IL-17 expression level and the severity of lupus nephritis, groups of IL-17^−/−^ mice and wild type (WT) mice were immunized with ALD-DNA. Eight weeks later, we analyzed the pathology of kidney in ALD-DNA immunized IL-17^−/−^ and WT mice. As shown in Figure 4A and B, ALD-DNA immunization effectively induced lupus nephritis in the WT mice. In contrast, we found that ALD-DNA failed to induce nephritis effectively in IL-17^−/−^ mice (Figure 4A and B, p<0.05). Further, we observed that ALD-DNA induced high levels of urine protein in the WT mice but not in IL-17^−/−^ mice (Figure 4C, p<0.05). To determine whether the immunization dose of ALD-DNA accounted for the impaired induction of lupus nephritis, groups of IL-17^−/−^ mice were immunized with an increasing dose of ALD-DNA. Results showed that the increased dose of ALD-DNA still could not induce the apparent lupus nephritis in IL-17^−/−^ mice (Figure 4D and E, p>0.05). These results demonstrated that IL-17^−/−^ mice were resistant to induction of lupus nephritis.

### IL-17 Expression Modified Immune Complex Deposition and Complement Activation in Kidney

**Figure 5:**
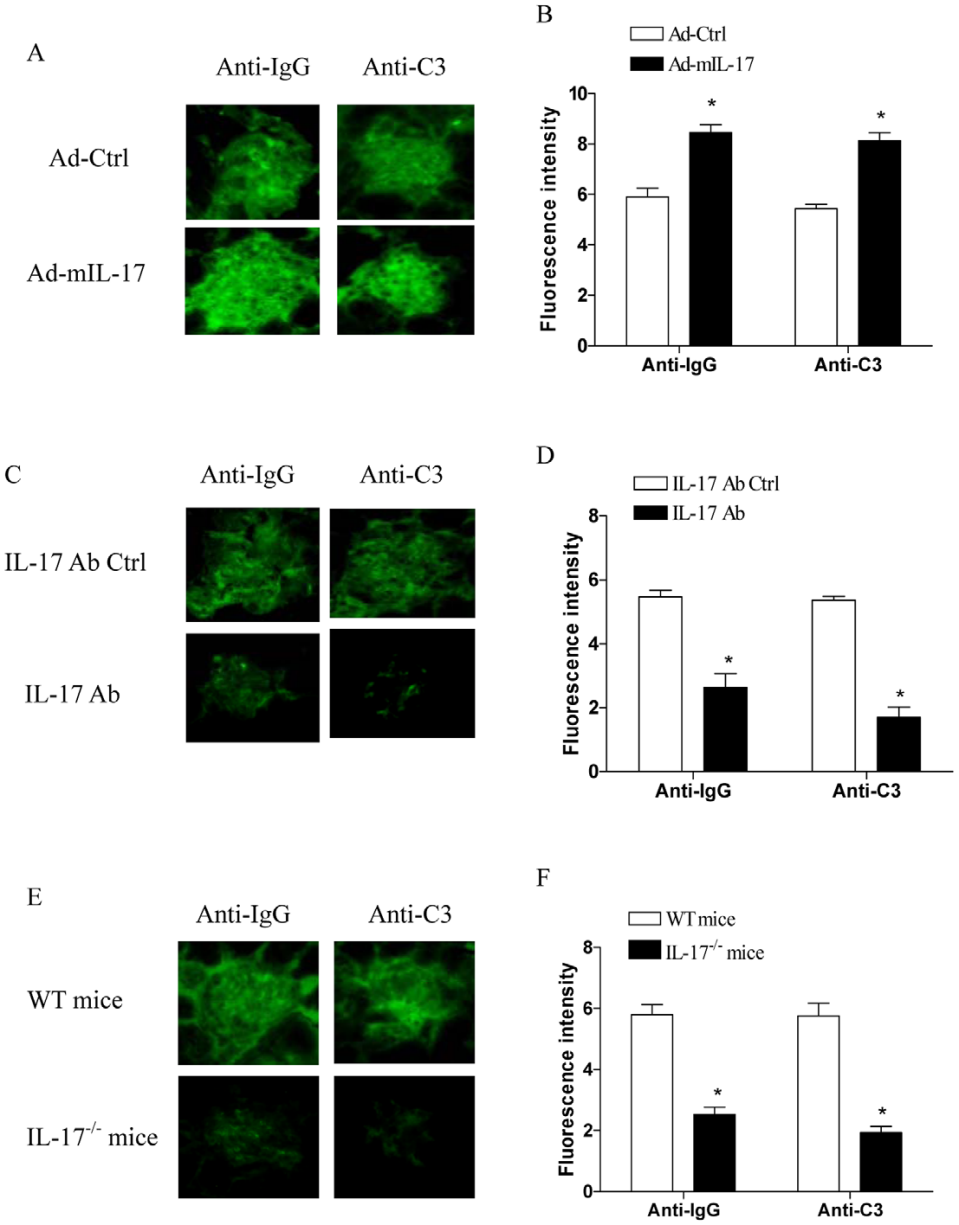
IL-17 was associated with immune complex deposition and complement activation in kidney. (A and B) Groups of BALB/c mice were treated with Ad-mIL-17 or Ad-Ctrl, and then immunized with 50 µg of ALD-DNA. Eight weeks later, the glomerular fluorescence was determined using anti-mouse IgG-FITC antibody or anti-mouse complement C3-FITC antibody. (C and D) Groups of BALB/c mice were injected with neutralizing antibody to IL-17 or the control antibody, and then immunized with 50 µg of ALD-DNA. Eight weeks later, the glomerular fluorescence was determined using anti-mouse IgG-FITC antibody or anti-mouse complement C3-FITC antibody. (E and F) Groups of IL-17^−/−^ mice or WT mice were immunized with 50 µg of ALD-DNA. Eight weeks later, the glomerular fluorescence was determined using anti-mouse IgG-FITC antibody or anti-mouse complement C3-FITC antibody. The fluorescence image shown was the representative result. The data of fluorescence intensity represented means (±SD) for eight mice in each group. *P<0.05.

In lupus nephritis, the pattern of glomerular injury is primarily related to the formation of the immune complex deposits in situ [2]–[5]. Therefore, to characterize the mechanisms account for the close correlation between IL-17 expression level and severity of lupus nephritis, kidney sections of ALD-DNA immunized mice were assayed for immune complex deposition and complement activation. As shown in Figure 5A and B, we showed that kidney sections from ALD-DNA immunized mice treated with Ad-mIL-17 exhibited elevated glomerular staining with anti-mouse IgG and anti-mouse complement C3 antibodies compared with the control group (p<0.05). Further, blockade of IL-17 significantly alleviated the glomerular staining with anti-mouse IgG and anti-mouse complement C3 antibodies in ALD-DNA immunized mice (Figure 5C and D, p<0.05). Consistently, we observed an impaired glomerular staining with anti-mouse IgG and anti-mouse complement C3 antibodies in IL-17^−/−^ mice (Figure 5E and F, p<0.05). In addition, we found no significant staining with anti-mouse IgG and anti-mouse complement C3 antibodies in mice treated with Ad-mIL-17 alone without ALD-DNA injection (data not shown). These results suggested that management of IL-17 expression level could modify the immune complex deposition and complement activation in kidney, which might account for its positive correlation with the severity of lupus nephritis.

### IL-17 was Crucial for Increasing Anti-dsDNA Antibody Production

**Figure 6:**
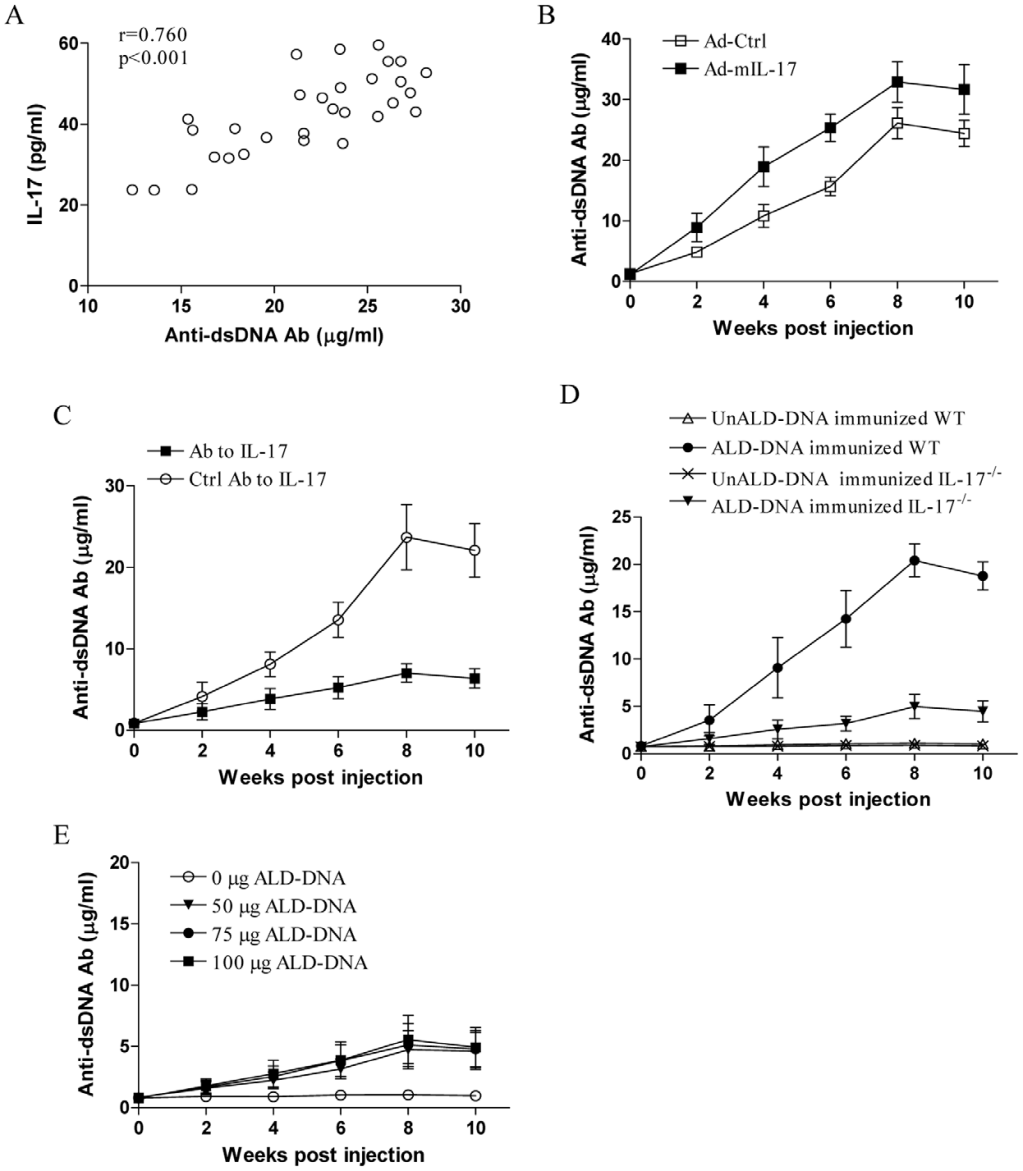
IL-17 increased anti-dsDNA antibody production. (A) Groups of BALB/c mice were immunized with 50 µg of ALD-DNA, and the correlation between serological level of IL-17 and anti-dsDNA antibody was analyzed in thirty two mice eight weeks post ALD-DNA injection. Each dot represented the average value for triplicate in each mouse. (B) Groups of BALB/c mice were treated with Ad-mIL-17 or Ad-Ctrl respectively, and then immunized with 50 µg of ALD-DNA. (C) Groups of BALB/c mice were injected with neutralizing antibody to IL-17 or the control antibody, and then immunized with 50 µg of ALD-DNA. (D) Groups of B6 WT mice or IL-17^−/−^ mice were immunized with 50 µg of the indicated DNA. (E) Groups of IL-17^−/−^ mice were immunized with the indicated dose of ALD-DNA. Data represented the means (±SD) for eight mice in each group. *P<0.05.

It is well acknowledged that anti-dsDNA antibody, which is closely correlated with the clinical syndrome and hence of diagnostic and even prognostic value, is an important pathogenic autoantibody involved in immune complex deposition that resulted in development of lupus nephritis [27]–[29]. Thus, to elucidate why management of IL-17 expression level could modify the immune complex deposition and complement activation in kidney, we explored the potential role of IL-17 in anti-dsDNA antibody production. As shown in Figure 6A, we found that the serum IL-17 expression level was closely correlated with the serological level of anti-dsDNA antibody in ALD-DNA induced lupus mice (p<0.05). Of important, we revealed that treatment with exogenous IL-17 increased anti-dsDNA antibody production, while in vivo blockade of IL-17 decreased anti-dsDNA antibody production (Figure 6B and C, p<0.05). Furthermore, we observed that ALD-DNA could not induce anti-dsDNA antibody effectively in IL-17^−/−^ mice (Figure 6D, p<0.05). When groups of IL-17^−/−^ mice were immunized with an increased dose of ALD-DNA, we found that increased dose of ALD-DNA still failed to induce a high level of anti-dsDNA antibody in IL-17^−/−^ mice (Figure 6E). These findings strongly demonstrated that IL-17 was crucial for increasing anti-dsDNA antibody production in lupus.

## Discussion

IL-17 is believed to be important for host defense against various pathogens, while its inappropriate/excessive production is considered to be involved in the development of inflammatory autoimmune diseases [30], [31]. In present study, we evaluated the association between IL-17 expression level and disease severity of lupus nephritis using ALD-DNA induced lupus model. We showed that IL-17 expression level was elevated and associated with the severity of lupus nephritis in ALD-DNA induced lupus mice. Of note, we found that treatment with exogenous IL-17 could enhance the severity of lupus nephritis, while blockade of IL-17 decreased the severity of lupus nephritis. In consistent, we found that IL-17^−/−^ mice were resistant to development of lupus nephritis. Combing our findings demonstrated that IL-17 expression level was closely and positively correlated with the severity of lupus nephritis, implicating a potential and promising approach of IL-17-based therapy against lupus nephritis. However, the mechanisms involved in the elevated expression of IL-17 in ALD-DNA induced lupus mice still needed successive studies.

Previous study showed that IL-17 could promote secretion of chemokines and other immune mediators from fibroblasts and epithelial cells, and thus may promote the recruitment of inflammatory cells to target organs including kidney [8]. In present study, we demonstrated the association of IL-17 expression level with immune complex deposition and complement activation in kidney. We showed that up-regulation of IL-17 enhanced the immune complex deposition and complement activation in kidney, while blockade of IL-17 alleviated the immune complex deposition and complement activation in kidney. We further confirmed this phenomenon and observed a weak intensity of immune complex deposition and complement activation in kidney of IL-17^−/−^ mice. These findings could account for the close correlation of IL-17 expression level with the severity of lupus nephritis, and suggested that IL-17 was indeed a promising target for treatment of lupus nephritis. Consistently, recent study showed that down-regulation of IL-17 production by T cells was correlated with the amelioration of murine lupus after treatment with either low-dose peptide tolerance therapy or nasal anti-CD3 antibody [32], [33]. However, it should be noted that a murine lupus model can not fully reproduce the complexity of clinical SLE in human patients. Further studies to reproduce our current findings in more clinically relevant models in primates and in clinical SLE patients were still needed. Of interest, two therapeutic human monoclonal antibodies against IL-17 (mAb AIN457 and LY2439821) have been developed and the clinical trials for uveitis, Crohn’s disease, psoriasis and rheumatoid arthritis are underway [34]–[36]. Thus, our present findings indicated that this therapeutic approach might be useful for patients with lupus nephritis.

Recent evidence suggested that IL-17 was an effective and important player in antibody response [25], [37]–[39]. IL-17 was responsible for the priming of collagen-specific T cells and IgG2a production, and thus the collagen-induced arthritis (CIA) was markedly suppressed in IL-17^−/−^ mice [40]. The antigen-specific Ig production was also significantly decreased in IL-17^−/−^ mice during allergic diseases such as methylated BSA-induced delayed-type hypersensitivity and ovalbumin (OVA)-induced airway inflammation [41]. In this study, we extended previous study by exploring the potential role of IL-17 in anti-dsDNA antibody production. We found that administration with exogenous IL-17 increased anti-dsDNA antibody production, while blockade of IL-17 decreased anti-dsDNA antibody production. Consistently, we observed an impaired generation of anti-dsDNA antibody in IL-17^−/−^ mice. Thus, here we reported for the first time that IL-17 was vital for generation of anti-dsDNA antibody in SLE. In addition, we also found that other autoantibodies including anti-Sm and anti-rRNP antibody also decreased substantially in IL-17^−/−^ mice compared with that in WT mice (data not shown), indicating a general effect of IL-17 on autoantibody production in SLE. Our results might partly explain the association of IL-17 expression level with the immune complex deposition and complement activation in kidney, and thus correlated with the severity of lupus nephritis. Besides, our data was also in line with previous study which showed that IL-17 could promote autoantibody production from peripheral blood mononuclear cells in patients with lupus nephritis [42]. In addition, recent study showed that IL-17 acted in synergy with B cell-activating factor to influence B cell survival, proliferation and differentiation into immunoglobulin-secreting cells [43], which might partly explain the crucial role of IL-17 in anti-dsDNA antibody production. However, the precise mechanisms for how IL-17 acted in generation of anti-dsDNA antibody still remain to be elucidated.

To conclude, here we demonstrated that IL-17 expression level was positively correlated with the severity of lupus nephritis. We did not focus on the proinflammatory effect of IL-17 in development of lupus nephritis. Instead, we reported the crucial role of IL-17 in anti-dsDNA antibody production, which might partly account for why IL-17 expression level was associated with the immune complex deposition in kidney and thus correlated with disease severity of lupus nephritis. Our findings provided a novel explanation through which IL-17 functioned in disease pathogenesis, and suggested that management of IL-17 expression level was a promising strategy for treatment of lupus nephritis.
